# Effects of Beetroot Juice Supplementation on Cardiorespiratory Endurance in Athletes. A Systematic Review

**DOI:** 10.3390/nu9010043

**Published:** 2017-01-06

**Authors:** Raúl Domínguez, Eduardo Cuenca, José Luis Maté-Muñoz, Pablo García-Fernández, Noemí Serra-Paya, María Carmen Lozano Estevan, Pablo Veiga Herreros, Manuel Vicente Garnacho-Castaño

**Affiliations:** 1 College of Health Sciences, University Alfonso X El Sabio University, Madrid 29651, Spain; rdomiher@uax.es (R.D.); jmatmuo@uax.es (J.L.M.-M.); pablgafe@uax.es (P.G.-F.); mloza@myuax.com (M.C.L.E.); pveigher@uax.es (P.V.H.); 2 Tecnocampus, College of Health Sciences, University of Pompeu Fabra, Mataró-Maresme, Barcelona 08302 Spain; educuen@hotmail.com (E.C.); nserra@tecnocampus.cat (N.S.-P.)

**Keywords:** nutrition, sport, exercise, nitric oxide, physical activity

## Abstract

Athletes use nutritional supplementation to enhance the effects of training and achieve improvements in their athletic performance. Beetroot juice increases levels of nitric oxide (NO), which serves multiple functions related to increased blood flow, gas exchange, mitochondrial biogenesis and efficiency, and strengthening of muscle contraction. These biomarker improvements indicate that supplementation with beetroot juice could have ergogenic effects on cardiorespiratory endurance that would benefit athletic performance. The aim of this literature review was to determine the effects of beetroot juice supplementation and the combination of beetroot juice with other supplements on cardiorespiratory endurance in athletes. A keyword search of DialNet, MedLine, PubMed, Scopus and Web of Science databases covered publications from 2010 to 2016. After excluding reviews/meta-analyses, animal studies, inaccessible full-text, and studies that did not supplement with beetroot juice and adequately assess cardiorespiratory endurance, 23 articles were selected for analysis. The available results suggest that supplementation with beetroot juice can improve cardiorespiratory endurance in athletes by increasing efficiency, which improves performance at various distances, increases time to exhaustion at submaximal intensities, and may improve the cardiorespiratory performance at anaerobic threshold intensities and maximum oxygen uptake (VO_2max_). Although the literature shows contradictory data, the findings of other studies lead us to hypothesize that supplementing with beetroot juice could mitigate the ergolytic effects of hypoxia on cardiorespiratory endurance in athletes. It cannot be stated that the combination of beetroot juice with other supplements has a positive or negative effect on cardiorespiratory endurance, but it is possible that the effects of supplementation with beetroot juice can be undermined by interaction with other supplements such as caffeine.

## 1. Introduction

Cardiorespiratory endurance is defined as a health-related component of physical fitness that relates to the ability of the circulatory and respiratory systems to supply fuel during sustained physical activity and to eliminate fatigue products after supplying fuel [1]. Cardiorespiratory endurance is a performance factor in all sports in which adenosine triphosphate (ATP) is resynthesized, mainly by aerobic metabolism or oxidative processes that produce energy. In these sports, the expended effort typically lasts longer than five minutes, primarily depending on the metabolic level of the oxidative processes involved [2]. Factors that limit performance in this type of endurance patterns include maximum oxygen uptake (VO_2max_), ventilatory thresholds (first and second ventilatory threshold) and energy efficiency or economy [3,4,5].

In competitive sports, 0.5%–1.5% improvements in performance are considered a critical difference [6]. In order to enhance the effects of training and improve performance, athletes often turn to nutritional supplements [7]. According to the American College of Sports Medicine (ACSM), adequate selection of nutrients and supplements, adjusting intake according to the exercise performed, is necessary for optimal performance in athletes [8]. However, not all supplements have been shown to produce a positive effect on performance. The Australian Institute of Sport [9], classified supplements to which athletes have access, with the goal of categorizing nutritional supplements based on the level of evidence for impact on an athlete's performance (Table 1). However, the effectiveness of supplements also depends on dosage and type of effort, because the potential ergogenic effect may differ by the specific type of sport [10].

Beetroot juice is used as a supplement because of its high inorganic nitrate (NO_3_^−^) content, a compound found naturally in vegetables and in processed meats, where it is used as a preservative [12]. 

**Figure 1:**
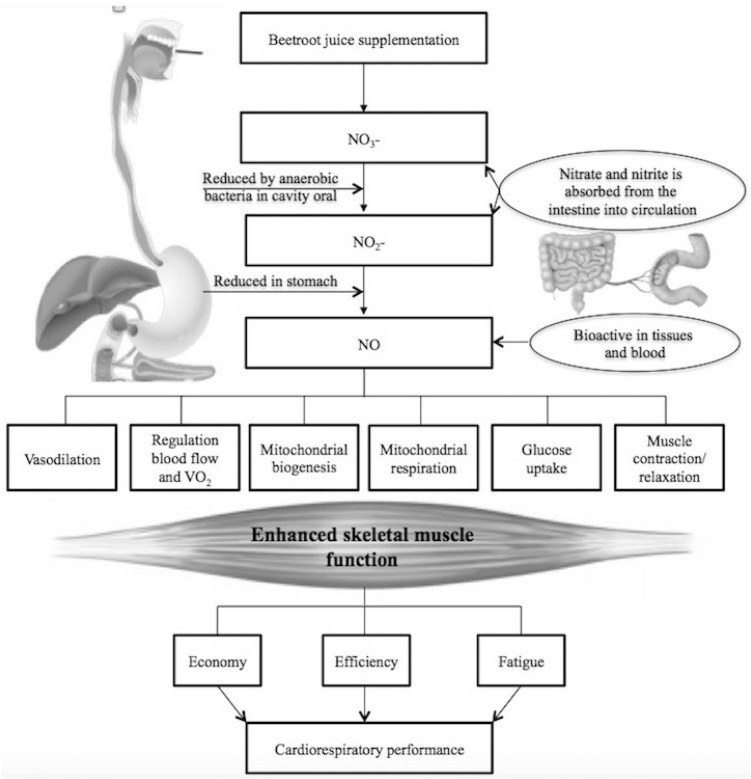
Pathway of nitric oxide (NO) production from beetroot juice supplementation. Nitrate (NO_3_^−^) is reduced to nitrite (NO_2_^−^) by anaerobic bacteria in the oral cavity and then to NO in the stomach. NO_3_^−^ and remaining NO_2_^−^ are absorbed from the intestine into the circulation, which can become bioactive NO in tissues and blood. NO induces several physiological functions improving skeletal muscle function and, consequently, increasing cardiorespiratory performance.

Once ingested, the NO_3_^−^ is reduced to nitrite (NO_2_^−^), by anaerobic bacteria in the oral cavity by the action of nitrate reductase enzymes [13] and then to nitric oxide (NO) in the stomach [14]. This physiological mechanism depends on the entero-salivary circulation of inorganic nitrate without involving NOS activity. Once in the acidic stomach, nitrite is instantly decomposed to convert to NO and other nitrogen oxides performing determinant physiological functions (Figure 1). Nitrate and remaining nitrite is absorbed from the intestine into the circulation, which can become bioactive NO in tissues and blood [14] under physiological hypoxia.

NO induces several physiological mechanisms that influences O_2_ utilization during contraction skeletal muscle. Physiological mechanisms for NO_2_^−^ reduction are facilitated by hypoxic conditions, therefore, NO (vasodilator) is produced in those parts of muscle that are consuming or in need of more O_2_. This mechanism would allow local blood flow to adapt to O_2_ requirement providing within skeletal muscle an adequate homogeneous distribution. This physiological response could be positive in terms of muscle function, although it would not explain a reduced O_2_ cost during exercise [15]. Another probable mechanism is related to NO_2_^−^ and NO as regulators of cellular O_2_ utilization [15]. 

In addition, a potent signaling molecule that affects cell function in many body tissues, NO is endogenously produced by synthesizing nitric oxide from l-arginine oxidation. The molecule has important hemodynamic and metabolic functions [16,17], being a major vasodilator that can increase blood flow to muscles [18] and promote oxygen transfer in the muscle. Additional physiological benefits of NO include improved mitochondrial efficiency and glucose uptake in muscle [19] and enhanced muscle contraction and relaxation processes [20]. Other researchers have reported that NO can act as an immunomodulator [21] and stimulates gene expression and mitochondrial biogenesis [22]. Given the positive effects of beetroot juice, which are induced by means of NO, this supplement has been proposed as part of the therapeutic approach in people with chronic obstructive pulmonary disease [23], hypertension [24], heart failure [25] and insulin resistance [26]. 

These findings reflect the importance of supplementation with NO_3_^−^ or nitrate salts to increase the bioavailability of NO in order to influence muscle function improving exercise performance, mainly in aerobic metabolism [27]. Therefore, supplementation with beetroot juice may have an ergogenic effect in athletes [9], especially with respect to cardiorespiratory endurance. However, the assumption that the beetroot juice supplementation improves performance in cardiorespiratory endurance under hypoxic conditions, and the combination of beetroot juice supplementation with other supplements, as caffeine, has a positive effect on cardiorespiratory endurance is controversial.

The objective of the present literature review was to analyze the effects of beetroot juice supplementation on cardiorespiratory endurance in several conditions (normoxia, hypoxia and beetroot juice with other supplements) and determine the appropriate dosage to enhance the potential ergogenic effects on performance. The focus of the article is mainly on the influence of beetroot juice of the acute and chronic responses on trained endurance athletes.

## 2. Methodology

A keyword search for articles published in English or Spanish since 2010 was carried out in the DialNet, MedLine, PubMed, Scopus and Web of Science databases on 8 June 2016. The search terms included beet, beetroot, nitrate, nitrite, supplement, supplementation, nutrition, “sport nutrition” and “ergogenic aids”. The 210 selected articles included at least one of those search terms, in combination with endurance, exercise, sport or athlete.

**Figure 2:**
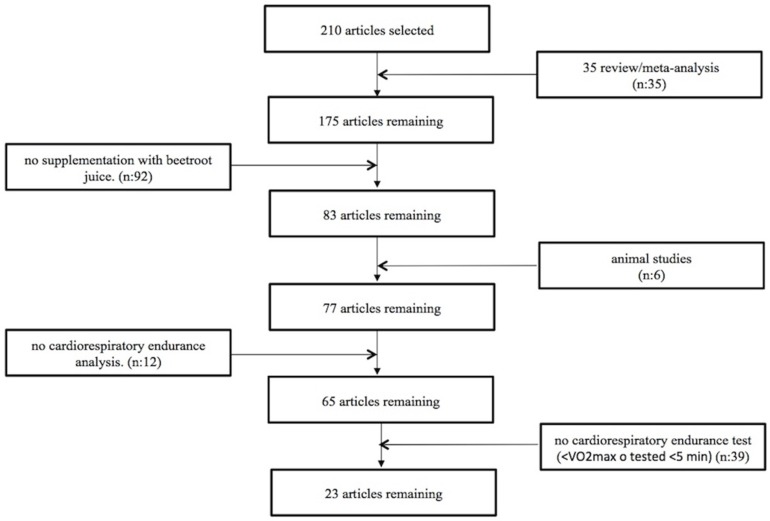
Flowchart of article selection.

Exclusion criteria were the following: literature reviews and meta-analyses, animal studies, population other than endurance athletes, and inadequate assessment of cardiorespiratory endurance, specifically defined as <VO_2max_ testing or no test lasting more than 5 min to determine how long the subject can maintain the lowest intensity at which VO_2max_ was achieved [28]. Therefore, 23 articles were selected for the present review (Figure 2). 

## 3. Results and Discussion

The selected studies on the effects of beetroot juice supplementation on cardiorespiratory endurance are summarized in Table 2.

In Table 2, 23 articles were examined regarding beetroot juice supplementation in normoxic conditions, hypoxic conditions and beetroot juice combined with caffeine supplementation: 11 of those articles were related to trained athletes, four of them to cyclists-triathletes, three to cyclists trained, two to trained kayakers, one to trained runners, one to trained swimmers, and one to healthy physically active people. Twenty-one of these articles assessed respiratory parameters including VO_2_ at several intensities (approximately 60%–100% VO_2max_, VT1)

Briefly, in trained athletes men and women in normoxia conditions appeared that beetroot juice supplementation enhances aerobic performance by a decrease in VO_2_ at several intensities (60%–100% VO_2max_, VT1) increasing the economy during exercise. In kayak studies, a decrease of VO_2_ at the same intensity in kayakers supplemented with beetroot juice compared to a placebo group was found. In trained swimmers, a decrease in energy expenditure in the experimental condition of beetroot juice supplementation was observed.

Regarding the supplementation with beetroot juice in hypoxic conditions, five studies were selected. The hypothesis that beetroot improves cardiorespiratory performance in hypoxic conditions is controversial.

Two studies evaluated the effect of the combination of beetroot juice and caffeine in men and women trained cyclists-triathletes, and one study evaluated the same supplementation in trained men athletes. The studies did not determine that the effects of beetroot juice combined with caffeine increase the cardiorespiratory performance regarding caffeine supplementation.

### 3.1. Acute Effects of Beetroot Juice Supplementation on Performance in Cardiorespiratory Endurance

Several studies have shown a positive effect of acute beetroot juice intake on various parameters of performance improvement associated with the cardiovascular and respiratory system. Economy is a parameter that expresses the relationship between oxygen consumption (VO_2_) and power generated or the distance traveled by an athlete [29], regarded as a performance factor in cardiorespiratory endurance [3,4,5]. Improved economy is due to achieving higher output power with the same VO_2_ level [30]. Another improvement attributed to beetroot juice supplementation is related to the increased blood flow, favoring the supply of oxygen to the mitochondria [50], which has the side effect of stimulating oxidative metabolism. In addition, supplementation with NO_3_^−^ could improve the processes of muscle contraction and relaxation [31].

A study in trained cyclists found that beetroot juice supplementation improves performance by 0.8% in a 50-mile test [32]. Significant increases in efficiency, measured as watts (W) per liter of VO_2_ (W/VO_2_) were observed in the last 10 miles; these improvements were associated with a decrease in time required to travel this distance. Another study [33] aimed to assess efficiency on a 40-min test at submaximal intensity (20 min at 50% VO_2max_ followed by 20 min at 70% VO_2max_). A decrease in VO_2_ and improved efficiency was also observed after beetroot juice supplementation, but did not reach statistical significance. After supplementation and immediately after the submaximal 40-min test, the time-to-exhaustion at an intensity of 90% VO_2max_ improved as much as 16% in the trained cyclists. These findings make us suspect that beetroot juice might have an ergogenic effect, increasing performance in prolonged cycling events that require alternations in relative intensity, from moderate to high VO_2max_, which is very characteristic of the stages of cycling races.

In a time trial of 16.1 km, supplementation with beetroot juice improved the performance of trained cyclists diminishing a completion time in a 2.7% and by 2.8% in a 4-km time trial [27]. Although, the protocol test used in this study had a high ecological validity, providing an accurate simulation of the physiological responses during competition, it is unclear that beetroot supplementation can increase the performance by this magnitude in elite cyclist [27].

This increased performance was also associated with W/VO_2_ improvements of 7% in a time trial of 16.1 km and 11% in 4-km time trial [27]. The observed improvements in efficiency match those found in high-performance kayakers when paddling at 60% relative VO_2max_ intensity or in a 4-min test [34].

Response to a submaximal VO_2_ test at constant load is very important to cardiorespiratory endurance in athletic performance. In this type of test, VO_2_ increases disproportionately during the first 3 min because of an increase at the respiratory center to meet the exercise-induced increase in energy demand [51]. At an intensity below VT1 60% VO_2max_ efforts, approximately stabilization of VO_2_ is observed from the 3-min point until the end of the effort [52]. Nonetheless, at intensities greater than VT1 a progressively greater recruitment of type II motor units occurs [53], which have a lower oxidative potential than type I [54], and therefore a progressive increase in VO_2_ is observed from the third minute until the end of the exercise. This has been called the slow component of VO_2_ [55], which has been identified as one of the main factors limiting performance in endurance exercise of moderate and/or high intensity [4], because the increase in the slow component of VO_2_ attains values of VO_2max_ at submaximal intensity, causing fatigue [56].

In experienced athletes, the effect of supplementation with beetroot juice (8.2 mmol nitrate) on time-to-exhaustion was tested at intensities of 60%, 70%, 80% and 100% peak power [35]. Athletes were able to maintain an intensity of 60% (Beetroot: 696 ± 120 vs. Placebo: 593 ± 68 s), 70% (Beetroot: 452 ± 106 vs. Placebo: 390 ± 86 s) and 80% (Beetroot: 294 ± 50 vs. Placebo: 263 ± 50 s) peak power significantly longer during exercise with supplementation, and there was a trend toward increased endurance at 100% peak power. The study results might reflect a lower VO_2_ response at submaximal intensities, which would reduce the increase in the slow component, delaying the time when the athletes reached VO_2max_ and therefore became fatigued. This would allow a longer sustained effort.

On the other hand, trained runners participating in a 5000-m test showed no significant overall improvement with beetroot juice supplementation, although they ran 5% faster in the later part of the race, particularly the last 1.1 miles [12]. The lack of significance could be related to the timing of the supplementation. Participants took the supplement 90 min before exercise; in the other studies cited, beetroot juice was provided 150–180 min before the effort [27,32,33,34,35] and ergogenic effects of supplementation with beetroot juice were observed at 150 min after ingestion [35].

### 3.2. Effects of Chronic Supplementation with Beetroot Juice on Cardiorespiratory Endurance

In addition to increasing blood flow and improving muscle contraction and relaxation, beetroot juice supplementation may improve the efficiency of mitochondrial respiration [50] and oxidative phosphorylation [57]. It seems, however, that acute supplementation is insufficient to produce mitochondrial biogenesis, suggesting that these adaptations may require longer supplementation protocols. In trained athletes, acute supplementation with beetroot juice for five days reduces VO_2_ as much as 3% at an intensity of 70% VO_2max_. The test was performed at 50% VO_2max_ for 10 min, followed by 10 min at 70% VO_2max_ [31]. Another study in trained cyclists confirmed that supplementation for a period of six days reduces VO_2_ in a 60-min test. The protocol consisted of 30 min at 45% VO_2max_ followed by another 30 min at 65% VO_2max_. In addition, riders were able to improve their 10-km time trial performance immediately following the submaximal test [30].

These studies clarify the benefits that could result from supplementation with beetroot juice in longer intake protocols of about six days, as was the case in the time-to-exhaustion test at submaximal intensities following acute supplementation [33,35]. Time-to-exhaustion improved at intensities of 70% of VO_2max_, between VT1 and VO_2max_ [37]. In trained swimmers, Pinna et al. [38] also corroborated the progressive ergogenic benefits of beetroot juice during an incremental test. At anaerobic threshold intensity, workload increased and aerobic energy expenditure decreased. 

In another study, in healthy subjects physically active but not highly trained in any particular sport, Vanhatalo et al. [36] evaluated the acute and chronic (15-day) effects of dietary supplementation with NO_3_^−^ on VO_2_ in a constant load test at an intensity of 90% of the gas exchange threshold (GET), similar to the anaerobic threshold, and in a progressive incremental ergometric cycle test, compared to controls. The peak power in the incremental test and the ratio of work rate to GET intensity were increased in the group that received the dietary NO_3_^−^ supplementation. The findings indicated that dietary supplementation reduces NO_3_^−^ oxygen consumption at submaximal exercise, and these effects can last for 15 days if supplementation is maintained.

Potential improvements observed in the anaerobic or lactate threshold intensity is especially important for athletes in various forms of endurance sports, because the level achieved in this parameter does not depend on motivation as it occurs when VO_2max_ is determined [58]. This threshold is considered a factor that better discriminates between cardiorespiratory endurance capacities than does VO_2max_ [2,58]. One of the physiological parameters that conditions improvement in the anaerobic threshold is increased mitochondrial population [59]. If the beetroot juice supplementation can promote mitochondrial biogenesis, we might assume that chronic supplementation with beetroot juice would decrease oxygen consumption at anaerobic threshold intensity as an adaptation to exercise.

It has also been suggested that additional beetroot juice supplementation may improve the muscle contraction functions. A study by Whitfield et al. [31] found that VO_2_ reduction after a constant load test at 70% VO_2max_ occurred without any changes in markers of mitochondrial efficiency such as adenine nucleotide translocase (ANT) and uncoupling protein 3 (UCP3). Similarly, other researchers have suggested that supplementation may positively affect the interaction of actin and myosin bridges [60] by modulating the release of calcium that occurs after the action potential [61]. The effects described by these authors indicate that supplementation with beetroot juice, whether acute or chronic, could improve performance in sports that are characterized either by a predominantly aerobic or anaerobic metabolism [38]. This could explain the positive effects on effort with a high prevalence of anaerobic metabolism observed in a 500-m kayak test [29] or in the contractile force developed by mice [62].

### 3.3. Effects of Beetroot Juice Supplementation on Performance in Cardiorespiratory Endurance under Hypoxic Conditions

Many competitions, such as the mountain stages in cycling, are held at high altitudes [39], where cardiorespiratory endurance is decreased relative to sea level [63]. Among the factors that could be responsible for this decrease, we would highlight decreased supply of oxygen to muscles, due to a partial reduction in oxygen pressure.

It is known that NO has an important role in the adaptation processes under hypoxic conditions; higher levels of NO_2_^−^ have been observed in Tibetans [18]. In a study of acute response to hypoxia, people who live at sea level who climb to high altitudes and show decreased NO levels have symptoms of acute altitude sickness [64,65]. The vasodilatory effects of NO may favor oxygen delivery [66], and supplementation with beetroot juice could be effective in reducing the ergolytic effects of hypoxia on cardiorespiratory endurance [39].

A recent study evaluated the effects of supplementation with acute and chronic beetroot juice on a 15-min test at an intensity of 50% VO_2max_ and a 10-km test carried out at a simulated altitude of 2500 m [40]. The test could not verify any positive effect of acute or chronic supplementation on any of the performance variables analyzed. In addition, studies have shown supplementation with beetroot juice did not improve performance in runners with a high level of training in an incremental intensity test or in a 10-km race [41] or in a 1500-m test or tests at various submaximal intensities (50%, 65% and 80% VO_2max_) [42]. The results in the latter study are also in line with those reported by McLeod [40]; in these two studies, beetroot juice was administered 90 and 120 min, respectively, before exercise. This may be an insufficient time interval for athletes to reach peak NO_2_^−^ levels in their bloodstream.

The results presented above conflict with other reports [39,43]. Kelly et al. [43] tested the effect of beetroot juice supplementation for three days on performance in a 5-min test at 80% VT1, followed by a test to the point of exhaustion at an intensity at 75% of VT1 and VO_2max_ and a simulated altitude of 2500 m. The results show that supplementation with beetroot juice reduced VO_2_ to 80% VT1 and there was a statistical trend to improvement in higher intensity exercise (*p* = 0.07). Improved efficiency was accompanied by a longer time-to-exhaustion in a test at 75% between VT1 and VO_2max_. In another study that simulated an altitude of 2500 m, supplementation with beetroot juice again reduced the VO_2_ during a 15-min test at 60% of VO_2max_ and increased the speed achieved in a 16.1-km time trial involving trained cyclists [39]. The results observed in the time trial were consistent with the improvements (2.8%) reported from a 4-km time trial after a protocol of acute supplementation with beetroot juice [27].

Masschelein et al. [44] found that six days of supplementation with beetroot juice can reduce VO_2_ at rest by 8%, and by 4% at 45% VO_2max_ intensity at a simulated altitude of 5000 m. Although the cited study is not directly generalizable to performance in various types of cardiorespiratory endurance, as competitions are unlikely to take place above an altitude of 2500 m, other parameters such as arterial oxygen saturation (SPO_2_) and deoxyhemoglobin (HHb) in muscle tissue were analyzed. The results showed that reductions in VO_2_ were accompanied by greater SPO_2_ and lower HHb after supplementation with beetroot juice, indicating decreased oxygen extraction by the muscle, which coincides with increased mechanical pedaling efficiency and lower levels of lactate in the blood.

Although the literature shows contradictory data, it is possible that supplementation with beetroot juice may effectively improve performance when hypoxia is present, because oxygenation would improve at the muscular level, reducing the ergolytic effects of hypoxia on aerobic performance.

### 3.4. Effects of the Combination of Beetroot Juice Supplementation with Other Supplements on Cardiorespiratory Endurance

Caffeine supplementation has become increasingly common among athletes [67]. Among its positive effects is increased stimulation of the central nervous system due to the antagonism of adenosine [68], increased catecholamines and contractility of skeletal muscle [69] that improves calcium output from the sarcoplasmic reticulum through the action potential [70], and a decrease in the subjective perception of pain and the regulation of thermoregulation [71]. Thus, caffeine supplementation has proven ergogenic effects on various modalities of cardiorespiratory endurance [72] and team sports [73,74]. A plateau effect occurs in performance improvement, at doses ranging from 3 to 6 mg/kg of caffeine [75]. To test whether the combined supplementation of beetroot juice (8 mmol of NO_3_^−^) and caffeine (5 mg/kg) had a greater effect than each supplement separately, researchers tested the corresponding study groups of cyclists tested for 30 min at 60% VO_2max_, followed by a test to exhaustion at 80% VO_2max_ [45]. Although the combined supplementation improved time to exhaustion VO_2max_ 80% by 46% compared to placebo, the improvement was insignificant. Furthermore, the additive effect of taking both supplements did not improve performance to a greater extent than separate supplementation with each one [46,47].

In a study that simulated the characteristics of an Olympic cycling time trial, the effect of supplementation in both men and women cyclists was tested using beetroot juice (8.2 mmol of NO_3_^−^) and caffeine (3 mg/kg) and the combination of both [47]. The only proven effects were that caffeine supplementation in combination with beetroot juice was effective in improving mean power and time trial results.

In a later study of trained cyclists and triathletes, performance was improved only in the athletes who received a caffeine supplement (3 mg/kg) [46]. No differences were observed in VO_2_. However, lactate concentration in the blood was increased when athletes received caffeine supplementation. Performance improvement was likely due to an increased anaerobic metabolism after caffeine intake; therefore, it is possible that the effects of supplementation with beetroot juice can be undermined by interaction with other supplements such as caffeine, which interferes with the effects of each supplement taken separately. 

### 3.5. Dosage

Peak NO_2_^−^ concentration in blood is obtained within 2–3 h of NO_3_^−^ supplementation [76] and the ergogenic effects of supplementation with beetroot juice can be observed at 150 min after ingestion [36]. Oral antiseptic rinses should not be taken with beetroot juice supplementation, as these can prevent the desired increase in NO_2_^−^ levels after NO_3_^−^ ingestion [77]. Although the majority of studies show ergogenic effects of beetroot juice at a supplementation dose of 6–8 mmol NO_3_^−^ (Table 2), it is possible that high performance athletes might require a slightly higher dose. For example, in high performance kayakers, the ergogenic effect of supplementation with beetroot juice was 1.7% in a 500-m test after ingestion of 9.6 mmol of NO_3_^−^ but a 4.8 mmol dose did not significantly improve results in a 1000-m test [29].

#### Practical Considerations

It appears that acute supplementation with beetroot juice increases the power output with the same VO_2_ levels [30]. This is an interesting finding for athletes as there is evidence that the economy is a key factor to improve cardiorespiratory performance increasing energy efficiency in endurance sports modalities. In addition, time to exhaustion at several intensities (60%–100% VO_2max_, MAP or VT1) is another usual performance parameter that is improved with acute beetroot supplementation [33,35]. However, not all studies show a positive effect to acute beetroot supplementation indicating that the efficacy of acute nitrate supplementation will be attributed to several factors such as the age, diet, physiological and training status, and other parameters as the intensity, duration, endurance modality and environment conditions [78]. Although most of the studies determine a supplementation dose of 6–8 mmol NO_3_^−^, it is unclear that this supplementation dose can be effective to improve cardiorespiratory performance in sports modalities such as kayaking or rowing. The dose should possibly be increased in sports modalities where muscular groups of upper limbs are implicated. Endurance athletes should take the dose of NO_3_^−^, approximately 90 min before the competition without oral antiseptic. Acute supplementation with beetroot juice is not sufficient to induce mitochondrial biogenesis, suggesting that mitochondrial adaptations could only occur after longer supplementation protocols. In chronic supplementations with beetroot juice, it appears that the benefits in cardiorespiratory performance might be produced in longer intake protocols of about six days [33,35]. Time-to-exhaustion at several intensities (between 70% and 100% VO_2max_, VT1) and the load at anaerobic threshold could be enhanced while aerobic energy expenditure could be diminished. Longer-term beetroot supplementation (15 or more days) could be effective, although it would be necessary other studies analyzing the mitochondrial biogenesis to corroborate whether mitochondrial adaptations depend on endurance training and/or beetroot supplementation. To date, this assumption is unknown.

The scientific literature shows discrepancies regarding the improvement of the cardiorespiratory performance induced by the supplementation of beetroot juice under hypoxic conditions. NO_3_^−^ could mitigate the ergolytic effects of hypoxia on cardiorespiratory in endurance athletes [39]. 

We cannot assert that the combination of beetroot juice with other supplements has a positive or negative effect on cardiorespiratory endurance. It is possible that the effects of supplementation with beetroot juice can be undermined by interaction with other supplements such as caffeine. More work is needed to confirm the results of these investigations.

## 4. Conclusions

- Acute supplementation with beetroot juice may have an ergogenic effect on reducing VO_2_ at less than or equal to VO_2max_ intensity, while improving the relationship between watts required and VO_2_ level, mechanisms that make it possible to enable increase time-to-exhaustion at less than or equal to VO_2max_ intensity.
- In addition to improving efficiency and performance in various time trials or increasing time-to-exhaustion at submaximal intensities, chronic supplementation with beetroot juice may improve cardiorespiratory performance at the anaerobic threshold and VO_2max_ intensities.
- Apparently, the effects of supplementation with beetroot juice might not have a positive interaction with caffeine supplementation, mitigating the effects of beetroot juice intake on cardiorespiratory performance, however, more work is needed to confirm the results of these investigations because the number of studies analyzing the effects of the combination of beetroot juice with other supplements, such as caffeine, is limited.
- Intake of beetroot juice should be initiated within 90 min before athletic effort, since the peak value of NO_3_^−^ occurs within 2–3 h after ingestion. At least 6–8 mmol of NO_3_^−^ intake is required, which can be increased in athletes with a high level of training.

## Figures and Tables

**Table 1:** Classification of nutritional supplements, based on performance effect. Adapted from Australian Institute of Sport [9] and Burke [11].

Category	Sub-Categories	Supplements
High level of evidence	Will improve athletic performance with adequate dosing and specific types of effort	β-alanine
		Sodium bicarbonate
		Caffeine
		Creatinine
		Beetroot juice
Moderate level of evidence	May improve performance, under specific dosing and effort conditions, although additional research is needed	Fish oils
		Carnitine
		Curcumin
		Glucosamine
		Glutamine
		HMB
		Quercetin
		Vitamins C and E
		Tart cherry juice
Low level of evidence	No demonstrated beneficial effects	Supplements not found in other categories
Prohibited supplements	May result in positive doping tests and therefore are prohibited	Substances on the list published annually by the *World Anti-Doping Agency* (WADA)

**Table 2:** Summary of studies that have evaluated the performance or metabolic responses after supplementation protocol with beet juice.

Reference	Participants	Experimental Conditions	Supplementation Protocol	Variables	Results
[12]	M (*n*: 5) and W (*n*: 6), trained athletes	EC1: beet juice, EC2: placebo	EC1: beet juice (8 mmol nitrate) (90 min before)	Test 5 km: Performance, HR, RPE	Performance: Last mile 1.1: faster EC1 vs. EC2 (5%), RPE: 1 mile: lower in EC1 vs. EC2
[27]	M, competitive cyclists (*n*: 9)	EC1: beet juice, EC2: placebo	EC1: 500 mL beet juice (6.2 mmol nitrate) (120 min before)	Tests 4 km and 16 km: respiratory parameters, performance	Performance in test 4 km: Time: lower in EC1 vs. EC2 (6.27 ± 0.35 vs. 6.45 ± 0.42 min), Power: Higher in EC1 vs. EC2 (292 ± 44 vs. 279 ± 51 W), W/VO_2_: Higher in EC1 vs. EC2 (93 ± 17 vs. 83 ± 9 W/L/min), Performance test of 16 km: Time: lower in EC1 vs. EC2 (26.9 ± 1.8 vs. 27.7 ± 2.1 min), Power: Higher in EC1 vs. EC2 (247 ± 44 vs. 233 ± 43 W), W/VO_2_: Higher in EC1 vs. EC2 (69 ± 3 vs. 64 ± 6 W/L/min)
[29]	M, national level athletes kayak (*n*: 5)	EC1: beet juice, EC2: placebo	Study A: EC1: 140 mL beet juice (4.8 mmol nitrate) (150 min before), Study B: EC1: 140 mL beet juice (9.6 mmol nitrate) (150 min before)	Study A: kayaking incremental test: Test 10 min (10 min + 5 min LT1 LT2) + 4 min test: respiratory parameters, lactate, performance (test 4 min), HR, RPE. Study B: Test 500 m	Study A: 4 min test: VO_2_: decreases in EC1 vs. EC2 (46.87 ± 2.56 vs. 47.83 ± 2.77 mL/kg/min), Economy: improved EC1 vs. EC2 (189.67 ± 8.17 vs. 193.90 ± 8.17 mL/kg/km). Study B: Test 500 m: Time: improved EC1 vs. EC2 (114.6 + 1.5 s vs. 116.7 + 2.2 s), Rowing often partially 100–400 m: increases in EC1 vs. EC2 (108 + 2 vs. 105 + 2 strokes), Partial speed 100–400 m: increases in EC1 vs. EC2 (4.40 + 0.03 vs. 4.30 + 0.05 m/s)
[30]	M, trained cyclists-triathletes (*n*: 13)	EC1: beet juice, EC2: placebo	EC1: beet juice 140 mL (8 mmol nitrate) (6 days)	Test 30 min at 45% MAP + 30 min at 65% MAP + test 10 km: respiratory parameters, lactate, glucose, performance (test to exhaustion at 80% VO_2max_), HR, RPE	Respiratory parameters VO_2_ at 45% MAP: lower in EC1 vs. EC2 (1.93 ± 0.05 vs. 2.0 ± 0.07 L/min), VO_2_ at 65% MAP: lower in EC1 vs. EC2 (2.94 ± 0.10 vs. 3.1 ± 0.09 L/min), Performance (test 10 km): improvement in EC1 vs. EC2 (953 ± 21 vs. 965 ± 21 s)
[31]	M, trained athletes (*n*: 13)	EC1: beet juice, EC2: placebo	EC1: 280 mL of beet juice (6.5 mmol nitrate) for 7 days. EC2: Control	Test 20 min (10 min to 10 min 50% + 70% VO_2max_): respiratory parameters	Respiratory parameters: 70% VO_2max_: oxygen consumption decrease in EC1 (3%)
[32]	M, trained cyclists (*n*: 8)	EC1: beet juice, EC2: placebo	EC1: 500 mL beet juice (6.2 mmol nitrate) (150 min before)	Test 50 miles: respiratory parameters, lactate, performance	Performance: last 10 miles: lower time in EC1 vs. EC2, W/VO_2_: higher in EC1 vs. EC2 (67.4 ± 5.5 vs. 65.3 ± 4.8 W/L/min)
[33]	M, trained athletes (*n*: 16)	EC1: beet juice, EC2: placebo	EC1: 450 mL beet juice (5 mmol nitrate) (115 min before)	Test 40 min [20 min at 50% VO_2max_ + 20 min at 70% VO_2max_] + time to exhaustion at 90% VO_2max_: respiratory parameters, performance (test to exhaustion at 90% VO_2max_), lactate, HR, RPE	Respiratory parameters: RER: greater in EC1 vs. EC2 at 50% VO_2max_ (0.89 ± 0.03 vs. 0.86 ± 0.06) and test to exhaustion (1.04 ± 0.06 vs. 1.01 ± 0.06), Performance (test to exhaustion at 90% VO_2max_): time increases in EC1 vs. EC2 (185 ± 122 s vs. 160 ± 109 s), Max lactate: Higher in EC1 vs. EC2 (8.80 ± 2.10 vs. 7.90 ± 2.30 mmol/L)
[34]	M, kayakers (*n*: 8)	EC1: beet juice, EC2: placebo	CE1: beet juice 70 mL (5 mmol nitrate) (180 min before)	Test 15 min at 60% MAP + 5 × 10 s. R: 50 s + 5 min recovery + Test 1 km kayak: respiratory parameters, performance (5 × 10 s) performance (1 km time trial), HR	Test 15 min at 60% MAP: respiratory parameters: VO_2_ lower in EC1 vs. EC2 (35.6 ± 2.5 vs. 36.8 ± 2.4 mL/kg/min), Test 1 km: respiratory parameters: VO_2_ lower in EC1 vs. EC2 (results not specified)
[35]	M, trained athletes (*n*: 9)	EC1: beet juice, EC2: placebo	EC1: 500 mL beet juice (8.2 mmol nitrate)	Tests to exhaustion at 60%, 70%, 80% and 100% VO_2max_: respiratory parameters, lactate, performance, HR	Performance: 60% VO_2max_: EC1 more time to exhaustion vs. EC2 (696 ± 120 vs. 593 ± 68 s), 70% VO_2max_: EC1 more time to exhaustion vs. EC2 (452 ± 106 vs. 390 ± 86 s), 80% VO_2max_: EC1 more time to exhaustion vs. EC2 (294 ± 50 vs. 263 ± 50 s)
[36]	M (*n*: 5) and W (*n*: 3), trained athletes (*n*: 8)	EC1: beet juice, EC2: placebo	EC1: beet juice 500 mL (5.2 mmol nitrate) (15 days)	Test 5 min at 90% VT 1 + incremental test: respiratory parameters, lactate, performance, HR, glucose	Respiratory parameters: Test 5 min at 90% VT1 (day 15): VO_2_ lower in EC1 vs. EC2 (1.37 ± 0.23 vs. 1.43 ± 0.23 L/min), Incremental test: Wpeak: Higher EC1 vs. EC2 (331 ± 68 vs. 323 ± 68 W), WVT1: Higher EC1 vs. EC2 (105 ± 28 vs. 84 ± 18 W)
[37]	M (*n*: 4) and W (*n*: 5), Healthy, physically active participants	EC1: beet juice, EC2: placebo	EC1: beet juice 140 mL (8 mmol nitrate) (6 days)	Test 4 min at 90% VT1 + test to exhaustion at 70% between VT1 and VO_2max_: respiratory parameters, lactate, performance (test to exhaustion at 70% between VT1 and VO_2max_), HR	Performance (test to exhaustion at 70% between VT1 and VO_2max_) higher EC1 vs. EC2 (635 ± 258 vs. 521 ± 158 s)
[38]	M, trained swimmers (*n*: 14)	EC1: beet juice, EC2: placebo	EC1: beet juice 500 mL (5.5 mmol nitrate) (6 days), EC2: placebo (6 days)	Incremental test in swimming	VT1: improvement in EC1 vs. EC2 (6.7 ± 1.2 vs. 6.3 ± 1.0 kg), energy expenditure: decreases in EC1 vs. EC2 (1.7 ± 0.3 vs. 1.9 ± 0.5 kcal/kg/h)
[39]	M, trained cyclists-triathletes (*n*: 9)	EC1: hypoxia (2500 m) + beet juice, EC2: hypoxia (2500 m) + placebo	EC1: beet juice 70 mL (5 mmol nitrate) (150–180 min before)	Test 15 min at 60% VO_2max_ + test of 16.1 km in hypoxia (2500 m): respiratory parameters, lactate, performance (16.1 km time trial)	Test 15 min at 60% VO_2max_: respiratory parameters: VO_2_ lower in EC1 vs. EC2 (improvement unspecified), performance (16.1 km time trial): Time: improved EC1 vs. EC2 (1664 ± 14 vs. 1716 ± 17 s), Power: improved EC1 vs. EC2 (224 ± 6 vs. 216 ± 6 W)
[40] *	M, trained cyclists (*n*: 11)	EC1: normoxia + beet juice, EC2: normoxia + placebo, EC3: hypoxia (2500 m) + beet juice, EC4: hypoxia (2500 m) + placebo	EC1: beet juice 70 mL (6.5 mmol nitrate) (120 min before), EC3: beet juice 70 mL (6.5 mmol nitrate) (120 min before)	Test 15 min 50% + test MAP 10 km: respiratory parameters, performance (10 km), HR	No differences in analyzed variables
[41]	M, trained runners (*n*: 10)	EC1: beet juice (n: 5), EC2: placebo (n: 5)	EC1: beet juice 70 mL (7 mmol nitrate) (150 min before)	Incremental test in hypoxia (4000 m). Test of 10 km in hypoxia (2500 m)	No differences between variables
[42]	M, trained athletes (*n*: 10)	EC1: acute beet juice, EC2: acute placebo, EC1: chronic beet juice, EC2: chronic placebo	EC1: 210 mL beet juice (6.5 mmol nitrate) (150 min before), EC2: placebo (150 min before), EC1: 210 mL beet juice (6.5 mmol nitrate) (8 days), EC2: placebo (8 days)	19 min test (7 min 50% VO_2max_ + 7 min at 65% VO_2max_ + 5 min at 80% VO_2max_) + test of 1500 m: respiratory parameters (test 19 min), performance (test 1500 m)	No significant differences between experimental conditions
[43] *	M, trained athletes (*n*: 12)	EC1: normoxia + beet juice, EC2: normoxia placebo, EC3: hypoxia (2500 m) + beet juice, EC4: hypoxia (2500 m)	EC1: beet juice 140 mL (8.4 mmol nitrate) (3 days), EC3: beet juice 140 mL (8.4 mmol nitrate) (3 days)	Test 5 min to 80% VT1 + 5 min to 75% between VT1 and VO_2max_ + time to exhaustion at 75% between VT1 and VO_2max_: respiratory parameters, performance, HR	Respiratory parameters (5 min at 80% VT1): VO_2_: lower in EC3 vs. EC4, performance (time to exhaustion at 75% between VT1 and VO_2max_): higher in EC3 vs. EC4 (214 ± 14 vs. 197 ± 28 s)
[44] *	M, trained athletes (*n*: 15)	EC1: normoxia + chronic beet juice, EC2: normoxia + placebo, EC3: hypoxia (5000 m) + chronic beet juice, EC4: hypoxia (5000 m) + placebo	EC1: beet juice 500 mL (0.7 mmol nitrate/kg) (6 days), EC3: 70 mL beet juice (0.7 mmol nitrate/kg) (6 days)	Test 20 min at 45% VO_2max_ + incremental test: respiratory parameters, lactate, performance, HR, RPE	Test 20 min at 45% VO_2max_: VO_2_: lower in EC3 vs. EC4 at rest (8%) and exercise (4%), Incremental test: time to exhaustion: higher EC2 vs. EC4 (527 ± 22 vs. 568 ± 23 s), Max. lactate: lower in EC1 vs. EC2 (9.1 ± 0.5 vs. 10.6 ± 0.3 mmol/L)
[45]	M, trained athletes (*n*: 14)	EC1: beet juice + caffeine, EC2: caffeine + placebo, EC3: beet juice + placebo, EC4: placebo	EC1: 140 mL beet juice (8 mmol nitrate) (90 min before) + 5 mg·kg^−1^ of caffeine (60 min before), EC2: 5 mg·kg^−1^ of caffeine (40 min before), EC3: 2 × 70 mL beet juice (8 mmol nitrate) (90 min before)	Test 30 min 60% + test to exhaustion at 80% VO_2max_: respiratory parameters, performance (test to exhaustion at 80% VO_2max_), HR, RPE, cortisol	RPE: lower at 15 min in test to exhaustion at 80% VO_2max_ in EC1 (17 ± 1) vs. EC2 (18 ± 1) and EC4 (19 ± 2)
[46]	W, trained cyclists and triathletes (*n*: 14)	EC1: beet juice + caffeine, EC2: caffeine + placebo, EC3: beet juice + placebo, EC4: placebo	EC1: beet juice 70 mL (7.3 mmol nitrate) (150 min before) + 5 mg·kg^−1^ of caffeine (60 min before), EC2: 5 mg·kg^−1^ of caffeine (60 min before), EC3: beet juice 70 mL (7.3 mmol nitrate) (150 min before)	Test of 20 km: respiratory parameters, lactate, performance, HR, RPE	Respiratory parameters: RER: EC2 vs. EC3 (+0.034) and EC4 (+0.033), lactate: EC2 vs. EC3 (+2.28 mmol/L) and EC4 (+2.04 mmol/L) and EC1 vs. EC3 (+2.74 mmol/L) and EC4 (+2.50 mmol/L), Performance: power: EC2 vs. EC3 improvement (+10.3 W) and EC4 (+10.4 W), time: improved EC2 vs. EC3 (+42.4 s) and EC3 (+45.1 s), HR: EC1 vs. EC2 vs. (+8.0 bpm), EC3 (+ 5.2 bpm) and EC4 (+ 6.5 bpm)
[47]	M (*n*: 12) and W (*n*: 12), trained cyclists-triathletes (*n*: 24)	EC1: beet juice + caffeine, EC2: caffeine + placebo, EC3: beet juice + placebo, EC4: placebo	EC1: 140 mL beet juice (8.4 mmol nitrate) (8–12 h before) + 3 mg·kg^−1^ of caffeine (60 min before), EC2: 3 mg·kg^−1^ of caffeine (40 min before), EC3: 140 mL beet juice (8.4 mmol nitrate) (8–12 h before)	Test of 43.83 km M and 29.35 km W: performance, HR, RPE	Performance: power: improvement in EC1 (258 ± 59 W) and EC2 (260 ± 58 W) vs. EC4 (250 ± 57 W), M time: improvement EC1 (1:02:38 ± 0:03:31 h:min:s) and EC2 (1:02:43 ± 0:03:04 h:min:s) vs. EC4 (1:03: 30 ± 0:03:16 h:min:s), W time: improving EC1 (0: 51: 1 ± 0:02:22 h:min:s) and EC2 (0:50:50 ± 0:02:56 h:min:s) vs. EC4 (0:51: 40 ± 0:02:31 h:min:s) in W
[48]	M, trained athletes (*n*: 22)	EC1: Beet juice (*n*: 11), EC2: placebo (*n*: 11)	6 weeks: EC1: 500 mL beet juice (5.8 mmol nitrate, approximately) + training in hypoxia (4000 m), EC2: placebo + hypoxia training (4000 m)	Progressive incremental test 30 min test: respiratory parameters, lactate, muscle glycogen, performance, HR	Incremental test: VO_2max_: improvement in EC1 60.1 ± 2.6 vs. 65.6 ± 2.1 L/min) and EC2 (60.8 ± 1.8 vs. 63.8 ± 1.6 L/min), HR_max_: EC1 vs. EC2 lower in (186 ± 3 vs. 197 ± 2 lpm), Max Lactate: EC1 vs. EC2 lower in (10.4 ± 0.7 vs. 11.8 ± 0.4 mmol/L), W at 4 mmol/L lactate: improvement in EC1 (215 ± 10 vs. 252 ± 9 W) and EC2 (204 ± 12 vs. 231 ± 10 W), 30 min test: performance: Pmean increases in EC1 (215 ± 10 vs. 252 ± 9 W) and EC2 (204 ± 12 vs. 231 ± 10 W)
[49]	M, trained athletes (*n*: 8)	EC1: beet juice, EC2: beet juice + Mouthwash with carbohydrates, EC3: placebo	EC1: 140 mL of beet juice (8 mmol nitrate) (150 min before), EC2: beet juice 140 mL (8 mmol nitrate) + mouthwash carbohydrates (150 min before)	Test 60 min at 65% VO_2max_: respiratory parameters, lactate, glucose, insulin, muscle glycogen, ATP, creatine	Lactate: increased EC1, EC2 and EC3. No differences between groups. Muscle glycogen: decline in EC1, EC2 and EC3. No differences between groups Creatine decline in EC1, EC2 and EC3. No differences between groups

ATP: adenosine triphosphate; EC: experimental condition; HR: heart rate; M: men; h: hours; kg: kilograms; km: kilometers; bpm: beats per minute; m: meter; min: minutes; mL: milliliter; MAP: maximal aerobic power; RER: respiratory exchange rate; RPE: subjective perception of effort; s: sec; VT1: first ventilatory threshold; VO_2_: oxygen consumption; VO_2max_: maximal oxygen consumption; W: Watt. All results presented reflect statistically significant differences (*p* < 0.05). * Studies where only the effect of beet juice vs. placebo in both hypoxic situations as compared normoxic condition.
